# Characterization and Determination of Nanoparticles in Commercial Processed Foods

**DOI:** 10.3390/foods10092020

**Published:** 2021-08-28

**Authors:** Jin Yu, Ye-Rin Jeon, Ye-Hyun Kim, Eun-Been Jung, Soo-Jin Choi

**Affiliations:** Division of Applied Food System, Major of Food Science & Technology, Seoul Women’s University, Seoul 01797, Korea; ky5031@swu.ac.kr (J.Y.); yrjeon0715@swu.ac.kr (Y.-R.J.); ye4978@swu.ac.kr (Y.-H.K.); ebe2@swu.ac.kr (E.-B.J.)

**Keywords:** top-down-approach-produced nanoparticle, nanobubble, nano-labeled processed foods, determination, particle size, surface chemistry

## Abstract

A wide variety of foods manufactured by nanotechnology are commercially available on the market and labeled as nanoproducts. However, it is challenging to determine the presence of nanoparticles (NPs) in complex food matrices and processed foods. In this study, top-down-approach-produced (TD)-NP products and nanobubble waters (NBWs) were chosen as representative powdered and liquid nanoproducts, respectively. The characterization and determination of NPs in TD-NP products and NBWs were carried out by measuring constituent particle sizes, hydrodynamic diameters, zeta potentials, and surface chemistry. The results show that most NBWs had different characteristics compared with those of conventional sparkling waters, but nanobubbles were unstable during storage. On the other hand, powdered TD-NP products were found to be highly aggregated, and the constituent particle sizes less than 100 nm were remarkably observed after dispersion compared with counterpart conventional bulk-sized products by scanning electron microscopy at low acceleration voltage and cryogenic transmission electron microscopy. The differences in chemical composition and chemical state between TD-NPs and their counterpart conventional bulk products were also found by X-ray photoelectron spectroscopy. These findings will provide basic information about the presence of NPs in nano-labeled products and be useful to understand and predict the potential toxicity of NPs applied to the food industry.

## 1. Introduction

Nanotechnology has been applied to a wide range of foods in the food industry to improve the stability, shelf-life, process convenience, quality, and nutritional values of processed foods [1,2,3]. Nanomaterials can be manufactured by two different methods, top-down and bottom-up approaches, and added as food additives, ingredients, and raw materials [4,5]. In the European Commission (EC), nanomaterials are defined as materials consisting of more than 50% of constituent particles in the size range of 1 to 100 nm based on number size distribution, including agglomerates or aggregates whenever the constituent particles belong to 1–100 nm [6]. Until now, most countries did not regulate the usage and labeling of nanomaterials in food products [7,8,9]. However, nanoparticles (NPs) have different properties compared with those of conventional bulk-sized particles (BPs), such as large surface area to volume ratios and high reactivity [10,11], which can affect their biological responses and toxicity [12,13]. Moreover, NPs are added in complex food matrices, leading to interactions between NPs and food components, which can also cause changes in the physicochemical properties of NPs [2,14,15]. Indeed, NPs can be decomposed into small molecules/ionic forms or form large aggregates with other molecules present in foods or biological systems, and thus NPs may not be further present in commercial products and in the body [2,16]. In this case, the toxicity evaluation of NPs can be followed according to relevant guidance for conventional materials [16]. The determination of the presence and fate of NPs in commercial processed foods is of importance to understand and predict their potential toxicity.

Many studies have focused on the characterization and toxicity evaluation of engineered inorganic NPs produced by bottom-up approaches. Indeed, the identification and fate determination of food additive silicon dioxide and titanium dioxide NPs in commercial food products were reported; silicon dioxide particles were found to be nano-sized aggregated particles of less than 100 m, whereas most titanium dioxide particles were determined to be larger than 100 nm [17,18,19,20]. Meanwhile, top-down approaches such as milling, grinding, homogenization, microfluidization, and high-speed rotary strike crushing are more generally applied to foods [21,22]. They are easy and effective methods to obtain nano-sized particles. However, the components of many functional and nutritional processed foods containing a variety of matrices cannot be exactly defined. Moreover, the most important nutritional components of foods are soft organic materials such as carbohydrates, proteins, lipids, vitamins, etc., which are incompatible with the conditions of electron microscopy, which is essential for size determination [16,22]. Hence, it is challenging to determine and characterize top-down-approach-produced (TD)-NPs in complex food products [16].

A wide range of commercial foods manufactured by top-down approaches are currently available on the market and labeled as nanoproducts [3,23]. Complex nano-powder products obtained by milling or grinding techniques of well-known conventional nutritional foods belong to this category [21]. Indeed, nanoproducts made from red ginseng, mushroom, soybean, ginko, grape seed, and lactic acid bacteria are on the market. These TD-NP products are aimed at increasing the oral absorption, bioavailability, and functional efficacy of active compounds related to nanosize [3,24,25]. On the other hand, waters containing nanobubbles have also attracted much attention. Nanobubble waters (NBWs) are produced by mixing gas (air, N_2_, H_2_, O_2_, and CO_2_) with water through different kinds of nanobubble generators, consequently having stable nanoscale bubbles suspended in the water [26]. NBWs have been reported to possess the potential to increase the seed germination rate [27,28,29], to promote the growth of plants [27,29], shellfish [30,31], and microorganism activity via fermentation [26], and to inhibit tumor cell development [32,33]. The functionality of NBWs was reported to be associated with the stability of nanobubbles in water, negative zeta potentials, and the generation of free radicals [26,30]. The mechanism of action of NBWs for seed and plant growing is still under investigation, but could be explained by the roles of stable nanobubbles (N_2_ and O_2_) in nutrient elements, thereby increasing metabolic activities [27,29].

The aim of this study was to characterize and determine the presence of NPs in nano-labeled processed foods. Commercially available processed foods with different manufactured forms, TD-NPs and NBWs, were chosen as representative powdered and liquid nanoproducts, respectively. The constituent particle sizes, size distributions, hydrodynamic diameters, surface chemistry, and the presence of NPs in commercial products were determined by applying and optimizing electron microscopy, dynamic electron microscopy (DLS), and X-ray photoelectron spectroscopy (XPS).

## 2. Materials and Methods

### 2.1. Materials

Seven commercial NBWs were purchased from different international manufacturers on the online market and numbered as NBW-1 to NBW-7 (Table S1). For comparative study, two different conventional sparkling waters (SWs) were supplied by a local company and numbered as SW-1 and SW-2. Seven powdered TD-NP foods indicated on product labeling were also obtained from different international manufacturers on the online market and numbered as NP-1 to NP-7 (Table S1). Two different conventional bulk-sized powdered foods manufactured by local companies were also purchased for comparative study and numbered as BP-1 (equivalent component to NP-1) and BP-2 (equivalent component to NP-2). The major components of NBWs and TD-NPs are listed in Table S1. All commercial foods were stored at 4 °C before analysis.

### 2.2. Sample Preparation

For powdered foods, a suspension (1 mg/mL) of each product was prepared by stirring in distilled water (DW) for 30 min, followed by sonication (160 Watts, Bransonic 5800, Branson Ultrasonics, Danbury, CT, USA) at 25 °C for 30 min (except inorganic-based NP-7 for 5 min) prior to experiments.

### 2.3. Dynamic Light Scattering and Electrophoretic Light Scattering Analysis

Hydrodynamic diameters and zeta potentials of nanobubbles in NBWs and NPs in powdered TD-NP foods were evaluated by DLS and electrophoretic light scattering (ELS), respectively, using a Zetasizer Nano System (Malvern Instruments, Worcestershire, UK). The hydrodynamic diameters of nanobubbles in NBWs were immediately measured after opening at 2 or 25 °C, and ELS analysis for all samples was performed at 25 °C. The stability of nanobubbles in NBWs was investigated by performing DLS and ELS analysis after storage at 4 °C for 6 months.

### 2.4. Scanning Electron Microscopic Analysis

The constituent particle sizes and shapes of powdered TD-NPs or their counterpart conventional BPs were determined by scanning electron microscopy (SEM; JSM-7800F Prime, JEOL, Tokyo, Japan). To observe TD-NP or conventional BP products as they are without any dispersion procedure, the powdered samples were directly placed onto a mount (SPECIMEN MOUNT, JEOL, Tokyo, Japan) with carbon tape (5 mm × 5 mm; E-SONG EMC, Seoul, Korea), and the excess powders were blown off with an air gun. On the other hand, 20 μL of the suspended samples after dispersion (stirring and sonication), as described in “2.1. Materials”, was dropped on the aluminum foil and dried at room temperature for 24 h. The aluminum foil loaded with the sample was attached to a mount with the carbon tape (5 mm × 5 mm). For all samples, the sample surface was coated with Pt/Pd via a sputtering process for 70 s. SEM images were obtained at 5–10 kV of low acceleration voltage [34,35,36]. The average particle sizes and size distributions were measured by randomly selecting more than 100 discrete particles from the SEM using ImageJ software (version 1.53a, National Institutes of Health, Bethesda, MD, USA).

### 2.5. Cryogenic Transmission Electron Microscopic Analysis

The constituent particle sizes and shapes of powdered TD-NPs or conventional BPs were determined by cryogenic transmission electron microscopy (cryo-TEM; Tecnai G2 Spirit TWIN, FEI, Hillsboro, OR, USA). The specimens for cryo-TEM analysis were prepared using an automated vitrification system (Vitrobot^®^; FEI, Eindhoven, The Netherlands) at 26 °C with a relative humidity of 100%. A droplet (3 μL) of the suspension as prepared in “Materials” was deposited on a lacey carbon film on a copper TEM grid and excess water was removed from the suspended sample by blotting with filter papers. Then, the grid was rapidly immersed in liquid ethane and transferred into liquid nitrogen. The vitrified specimens were observed at an accelerating voltage of 120 kV. The average particle sizes and size distributions were measured by randomly selecting more than 100 discrete particles from the cryo-TEM images using ImageJ software (version 1.53a, National Institutes of Health, Bethesda, MD, USA).

### 2.6. Surface Chemical Analysis

The surface chemical analysis for powdered TD-NPs or conventional BPs was performed by XPS (K-Alpha XPS, Thermo Fisher Scientific, West Palm Beach, FL, USA) using Al-Kα X-ray source with a nominal spot size of 200 μm. The powdered samples were fixed on a sample holder using conductive carbon tape. Survey spectra were obtained at 200 eV pass energy and 1.0 eV energy step of the analyzer and recorded from 1350 to 0 eV. The individual high-resolution spectra for C1s, O1s, and N1s were recorded at 40 eV pass energy and 0.05 eV energy step. The obtained high-resolution spectra were fitted using Igor Pro software (version 8.04, Wavemetrics, Lake Oswego, OR, USA).

### 2.7. Statistical Analysis

Results were presented as means ± standard deviations. A one-way analysis of variance (ANOVA) with Tukey’s test was performed using SAS version 9.4 (SAS Institute Inc., Cary, NC, USA) to determine the significances of intergroup differences. Statistical significance was accepted for *p* values of less than 0.05.

## 3. Results

### 3.1. Characterization of Nanobubbles in NBWs

The presence of nano-sized bubbles in NBWs and their size distributions were determined by measuring the hydrodynamic diameters. Table 1 shows that polydispersity index (PDI) values for all products, except NBW-2, were higher than 0.9, and unstable DLS histograms were observed when DLS analysis of NBWs and SWs was performed at 25 °C. Thus, the analysis was further carried out at 2 °C because bubbles are generally stable at refrigeration temperature. The results show that NBW-2 and NBW-4 had 100% of particle fractions larger than 200 nm, and a portion of particle fractions ranging from 100 to 200 nm were present in NBW-1, NBW-5, and NBW-7 (Table 2).

The Z-average diameters of nanobubbles in all NBWs were larger than 300 nm, considering that the PDI values ranged from 0.2 to 0.5. In the case of NBW-3 and NBW-6, the PDI values were 0.9 and 1.0, respectively, as high as observed in DW, and DLS histograms were not stable. Conventional SWs showed unstable DLS histograms and PDI values of 1. The Z-average diameters of SW-1 and SW-2 were determined to be more than 30,000 nm. On the other hand, the ELS results indicate that the zeta potentials of all NBWs were negative and ranged from −20 to −5 mV, whereas zeta potentials of SW-1 and SW-2 were close to 0 mV. 

The stability of NBWs was checked after storage at refrigeration temperature for 6 months by performing DLS analysis. Table 3 demonstrates that the Z-average diameters and PDI values increased compared with those in Table 2, and unstable DLS histograms were observed for all products, except NBW-2 and NBW-5. The zeta potential values for all NBWs were negative after storage for 6 months but changed to less negative charges compared with those in Table 2, except NBW-2 and NBW-5.

### 3.2. Characterization of Powdered TD-NP Foods

**Figure 1:**
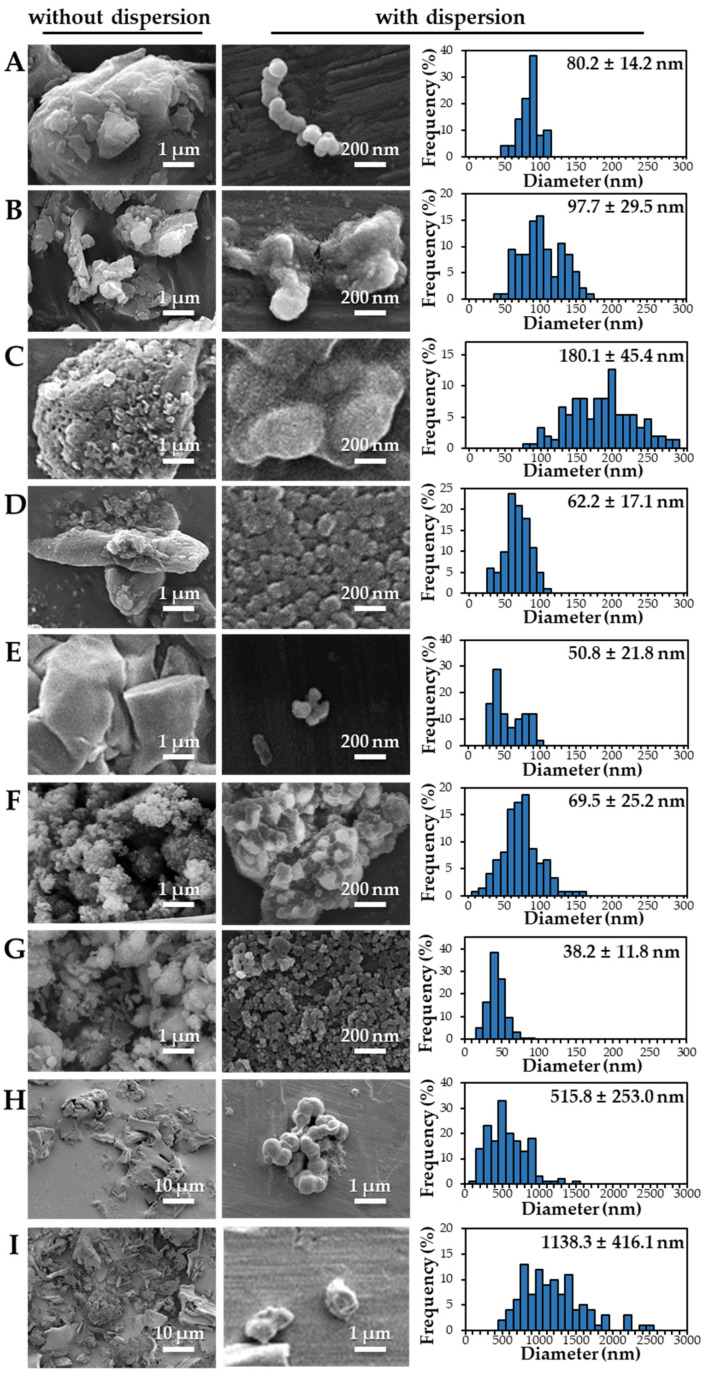
Scanning electron microscopy (SEM) images and size distributions of TD-NPs or conventional bulk-sized BP-1 and BP-2 composed of the same components as NP-1 and NP-2, respectively. (**A**) NP-1, (**B**) NP-2, (**C**) NP-3, (**D**) NP-4, (**E**) NP-5, (**F**) NP-6, (**G**) NP-7, (**H**) BP-1, and (**I**) BP-2. The size distributions of constituent particles were determined by randomly selecting more than 100 particles from the SEM images. Abbreviations: TD-NP, top-down-approach-produced nanoparticle; BP, bulk-sized particle; NP, nanoparticle.

The size distributions and morphology of TD-NPs were determined by SEM with/without dispersion. Comparative study with conventional BP-1 and BP-2 composed of equivalent components to NP-1 and NP-2, respectively, but conventionally produced, was also performed. Counterpart conventional BPs were not available on the market for other TD-NPs. The SEM analysis was carried out at a low voltage of 5–10 kV because most commercially available TD-NP products contain many organic matrices, except NP-7, and these organic matrices are sensitive to irradiation by electrons [34,35,36]. Figure 1 demonstrates that TD-NPs were highly aggregated without any dispersion. More dispersed but still aggregated particles were observed after stirring and sonication in all cases. The average particle sizes of all TD-NPs were less than 100 nm except NP-3, as determined by randomly selecting at least 100 particles from the SEM images. The relatively small constituent particle size of NP-7 consisting of an inorganic matrix was found.

On the other hand, large constituent particle sizes of conventional BP-1 and BP-2 compared with those of NP-1 and NP-2 were examined even after stirring and sonication, showing average sizes of about 516 and 1138 nm for BP-1 and BP-2, respectively.

The DLS results reveal that the Z-average diameters of TD-NPs dispersed in DW ranged from 327 to 2148 nm, indicating their aggregate states under aqueous conditions (Table 4). The particle fractions of all TD-NPs were larger than 200 nm, except NP-6 (93% of particle fraction less than 100 nm). Meanwhile, the Z-average diameters of conventional BPs were also larger than 1000 nm, with 100% of particle fractions larger than 200 nm. The ELS results demonstrate that the zeta potential values for all powdered nano and bulk products were negative.

### 3.3. Constituent Particle Sizes of Powdered TD-NP Foods

**Figure 2:**
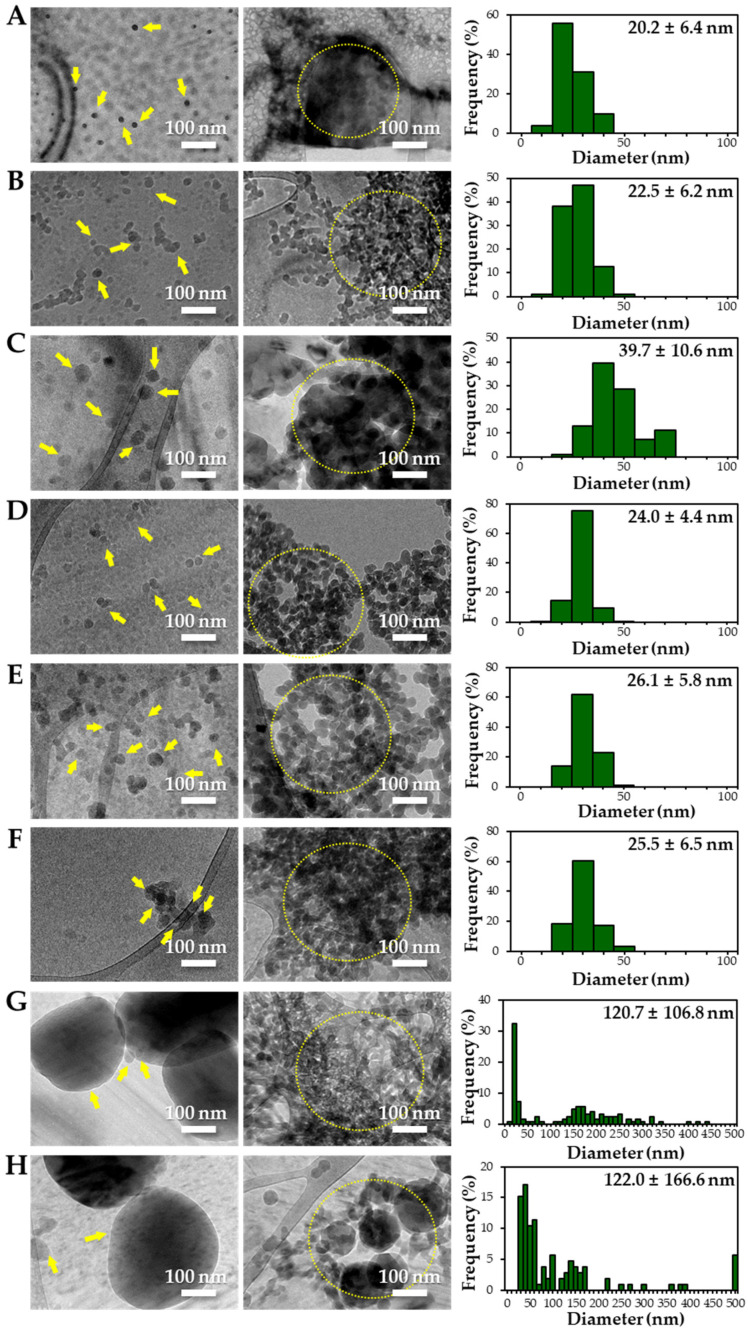
Cryogenic transmission electron microscopy (cryo-TEM) images and size distributions of TD-NPs or conventional BP-1 and BP-2 composed of the same components as NP-1 and NP-2, respectively. (**A**) NP-1, (**B**) NP-2, (**C**) NP-3, (**D**) NP-4, (**E**) NP-5, (**F**) NP-6, (**G**) BP-1, and (**H**) BP-2. Yellow arrows and yellow dotted lines indicate separated individual particles and aggregated forms, respectively. Particle size distributions were determined by randomly selecting more than 100 particles from the cryo-TEM images. Abbreviations: TD-NP, top-down-approach-produced nanoparticle; BP, bulk-sized particle; NP, nanoparticle.

The constituent particle sizes and shapes of TD-NPs consisting of organic matrices (NP-1 to NP-6) and conventional BPs were further examined by cryo-TEM after dispersion (stirring and sonication). Cryo-TEM is a powerful tool to determine the structure, size, and shape of soft organic materials incompatible with the conditions of electron microscopic measurements [34,37]. This is based on ultra-fast cooling and conversion of a liquid sample to a vitrified glassy specimen, which permits TEM analysis without significant morphological changes [37]. The results show that the average sizes of constituent particles of all TD-NPs were smaller than those observed by SEM (Figure 1), showing ~20 to 40 nm (Figure 2). The particles were present as both individual separated particles and aggregated forms in all cases. Rounded or irregular particle shapes were observed depending on material types. On the other hand, conventional BP-1 and BP-2 had larger average sizes (larger than 100 nm) and broader size distributions of constituent particles than those of NP-1 and NP-2, respectively.

### 3.4. Surface Chemical Characterization of Powdered TD-NP Foods

**Figure 3:**
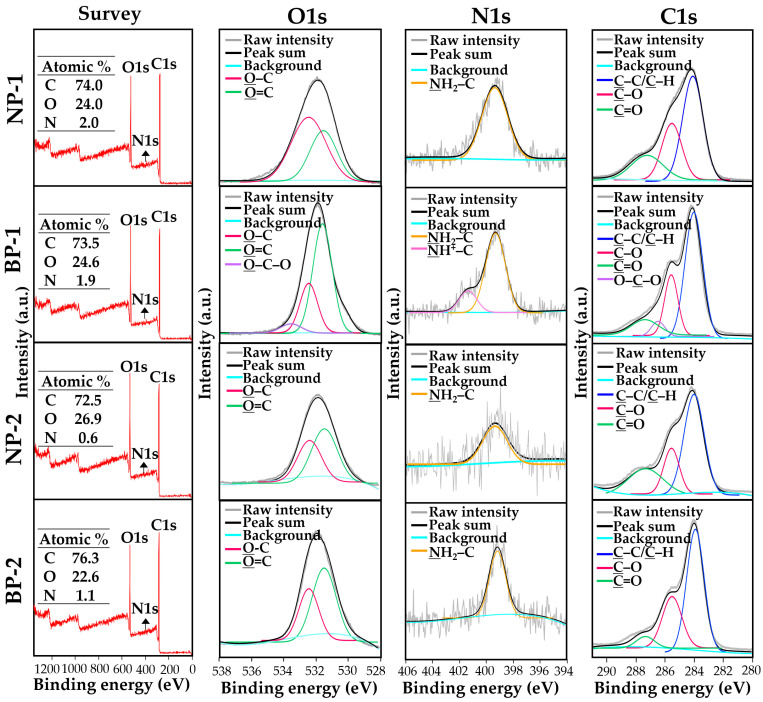
X-ray photoelectron spectroscopy (XPS) survey and high-resolution spectra (O1s, N1s, and C1s) of TD-NPs (NP-1 and NP-2) or conventional BPs (BP-1 and BP-2). Abbreviations: TD-NP, top-down-approach-produced nanoparticle; BP, bulk-sized particle; NP, nanoparticle.

XPS analysis was performed to compare the elemental composition and chemical state of NP-1 and NP-2 with those of conventional BP-1 and BP-2. The spectra survey shows that peaks at 532, 399, and 284 eV correspond to O1s, N1s, and C1s, respectively (Figure 3). Based on these spectra, the chemical compositions (%) of three main elements (O, N, and C) between NP-1 and BP-1 were highly similar, whereas slightly different elemental compositions between NP-2 and BP-2 were found. When spectra of each element were examined, O1s spectra of NP-1, NP-2, and BP-2 were deconvoluted into two peaks attributed to structural bonds of O=C at 531.4 eV and O–C at 532.4 eV, whereas a peak of O–C–O at 533.4 eV, together with O=C and O–C bonds, was determined only in BP-1. Intensity changes in O=C and O–C between NP-1 and BP-1 were also remarkably found, which was not observed between NP-2 and BP-2. The peaks of N1s spectra for NP-1, NP-2, BP-1, and BP-2 correspond to the NH_2_–C bond at 399.3 eV, and a peak of NH^+^–C at 401.36 eV was detected only in BP-1. In the case of C1s, the spectra for all samples were commonly deconvoluted into three peaks at 284.1, 285.6, and 287.4 eV, corresponding to C–C/C–H, C–O, and C=O bonds, respectively. Meanwhile, a peak of O–C–O at 286.5 eV was only identified in BP-1. Intensity increases in C–C/C–H and decreases in C=O bonds in BPs compared with NPs were also observed.

## 4. Discussion

In this study, we characterized commercially available nano-labeled powdered and liquid foods, such as TD-NPs and NBWs, and the presence of NPs in processed foods was determined. The characterization of NPs in commercial products is of importance because diverse organic matrices in foods disturb the analysis of particle size using electron microscopy, and NPs have high reactivity compared with micro-sized materials. Moreover, the determination of NPs in commercial foods is crucial not only for the regulation of nano-labeled products, but also for the safety evaluation of NPs. If NPs are not present or completely decomposed in final products, nano-labeling is not allowed, and the toxicity evaluation of new nanomaterials is not mandatory or can be followed according to classical methods for conventional bulk-sized materials [16].

The DLS results show that the Z-average sizes measured at 2 °C were much smaller (Table 2) than those measured at 25 °C (Table 1), when the sizes with reliable PDI values less than 0.7 were considered [38]. This can be explained by the high stability of gases at a low temperature [39,40]. The instability of bubbles was also clearly shown in the DLS histogram in Table 1 and in NBW-3 (Table 2). In all cases, no particle fractions less than 100 nm were observed and a small portion of fractions between 100 and 200 nm was found only in NBW-1, NBW-5, and NBW-7 (Table 2). It is worth noting that 100% of fractions less than 100 nm were detected in DW, but they cannot be considered as NPs due to the PDI value of 1.0 and unstable DLS histogram (Table 2). Hence, it seems that nanobubbles were not present in NBW-6, SW-1, and SW-2. The Z-average sizes and PDI values of all nanobubbles measured after 6 months increased, except NBW-2 and NBW-5 (Table 3), implying the instability of nanobubbles in NBWs during storage. The fact that the zeta potential values for all NBWs after 6 months, except NBW-2 and NBW-5, changed to less negative charges compared with those in Table 2 also supports the instability of nanobubbles in most NBWs (Table 3). All the results indicate that nanobubbles are not stable in most commercially available NBWs, and it is possible that nanobubbles less than 100 nm are not present at a commercially available stage. On the other hand, the Z-average diameters of bubbles in conventional SWs were much larger than those in NBWs under the same conditions, and the differences in zeta potentials between NBWs and SWs were clearly found (Table 2). These results suggest that NBWs have different physicochemical characteristics compared with those of conventional SWs, although no particles less than 100 nm were found in NBWs. Nevertheless, two among seven samples tested (NBW-2 and NBW-5) had stable PDI values, DLS histogram, zeta potential values, and Z-average diameters after 6 months. Hence, the manufacturing process applied to NBWs seems to differ from the conventional one. It is probable that nanobubbles are present at an initial stage just after production, but manufacturing technique, storage, and distribution conditions can be different from manufacturers, which affects the stability of nanobubbles. Further study to enhance the stability of nanobubbles in NBWs is required for nano-labeled products.

Our SEM results clearly show that the average sizes of powdered TD-NPs tested were less than 100 nm, except NP-3, but they formed agglomerates or aggregates even after stirring and sonication (Figure 1). When the SEM images with/without dispersion are compared, it is clear that NP dispersion, such as stirring and sonication, is necessary to examine nano-sized particles in powdered TD-NP foods. Aggregate/agglomerate fates of TD-NPs were also confirmed by DLS results, except NP-6 (Table 4). Meanwhile, broader particle size distributions with larger average sizes (~516 to 1138 nm) of conventional BPs than those of TD-NPs were examined (Figure 1), indicating the difference in particle sizes between BPs and TD-NPs. It is worth noting that the SEM analysis was performed at a low acceleration voltage (5–10 kV) due to high contents of organic matrices in all samples, except inorganic-based NP-7 [34,35,36].

More clear images on size distributions, constituent particle sizes, and shapes could be obtained by cryo-TEM analysis performed after vitrification, showing the presence of NPs less than 100 nm, but aggregated fates in all organic-based powdered TD-NP foods (Figure 2). The average sizes of constituent particles ranged from ~20 to 40 nm (Figure 2), smaller than the sizes measured by SEM (Figure 1). The discrepancy in size between SEM and cryo-TEM analysis may be related to the preparation procedure for cryo-TEM specimens. Indeed, both SEM and cryo-TEM analysis were carried out after the stirring and sonication of the samples, but a further vitrification procedure was carried out for cryo-TEM specimens, and TEM analysis was performed under cryogenic conditions. Cryo-TEM involves an ultra-fast conversion of the state of the material from fluid to glassy without adding other compounds, thereby contributing to maintaining the intact composition or structure of the material [37]. These results indicate that stirring and sonication generally applied for electron microscopic analysis may not be enough to disperse TD-NPs agglomerated/aggregated with multi-components present in processed foods. Therefore, cryo-TEM analysis can be an effective approach to determine organic-based NPs in complex food systems. NP-3 had a larger constituent particle size than others by SEM (Figure 1) and cryo-TEM (Figure 2) analysis, implying the effect of matrix types or manufacturing process on the characteristics of TD-NPs. On the other hand, large average sizes (~120 nm), broad size distributions up to 500 nm, and high aggregates of conventional BPs compared with those of TD-NPs were found by cryo-TEM, supporting the presence of NPs in TD-NP products. Taken together, NPs were present in powdered TD-NP products by SEM and cryo-TEM analysis, although they formed high agglomerates or aggregates. It is worth noting that agglomerated or aggregated particles can exhibit the same property as unbound NPs when they are released from the agglomerates or aggregates under environmental and biological conditions, and thus they are included in the category of NPs [6]. Moreover, the number size distribution threshold of 50% in the size range 1–100 nm for NP definition may be replaced by a threshold between 1 and 50% in specific cases where concerns for the environment, health, and safety are warranted [6,16]. Therefore, it can be concluded that the powdered TD-NP products tested contain nano-sized agglomerated/aggregated particles. It is possible that TD-NPs have different toxicity compared with conventional BPs, and thus further study on biological responses of TD-NPs is required to be performed to ascertain their potential toxicity.

The XPS results demonstrate the differences in chemical state between NP-1 and BP-1 as well as in elemental composition between NP-2 and BP-2 (Figure 3). It is probable that top-down-based processing such as milling and grinding leads to the decomposition or formation of chemical bonds. In actual states, it is difficult to exactly explain the reason why such changes occur in TD-NP products, since they have various components including nutrients and functional ingredients. However, the remarkable changes in chemical bonds between NP-1 and BP-2 may be related to the poor stability of ginsenosides, the main functional components of ginsengs, against processing conditions, such as pH, temperature, heat, and extract solvents [41]. The slightly different elemental compositions between NP-2 and BP-2 are more likely to be related to the compositions of raw materials or the degradation of certain compounds during TD-NP processing. These results suggest that elemental compositions and chemical bonds could be affected by top-down processing for NP products. The degree of chemical change may be associated with compositions of food matrices and processing methods. Surface chemical characterization using XPS can be a useful tool to differentiate NPs from conventional BPs. Further study on more extended samples is required to elucidate the mechanism involved in elemental and chemical changes. Moreover, the effect of food matrix types on the characteristics and safety aspects of NPs in commercial foods should also be elucidated. 

## 5. Conclusions

In this study, the characterization of NPs in commercially available powdered and liquid foods was carried out and the presence of NPs was determined. The different characteristics between nanobubble waters and conventional sparkling waters were confirmed by Z-average diameters and zeta potential values. However, nanobubbles in most nanobubble waters tested were not stable during storage, and the presence of NPs less than 100 nm was not confirmed. On the other hand, constituent particles of less than 100 nm were clearly observed in powdered top-down-approach-produced NP products compared with those of conventional bulk-sized particles by SEM at a low acceleration voltage and cryo-TEM analysis, but they were present as agglomerated or aggregated forms. The differences in chemical composition and chemical state between top-down-approach-produced NPs and conventional bulk-sized particles were also found, suggesting a possible change in surface chemistry during top-down-approach processing. These findings will provide crucial information about the presence of NPs in nano-labeled products and be useful to understand and predict the potential toxicity of TD-NP foods. Further extended study on a wide range of nano-labeled foods is required to elucidate the mechanism involved in their characteristic changes. Moreover, the toxicity evaluation of nano-labeled products must be performed to ensure the safety of NPs in the food industry.

## Figures and Tables

**Table 1:** Particle fractions and hydrodynamic diameters of bubbles in NBWs or in conventional SWs at 25 °C ^1^.

Sample	Fraction (Number%)			Z-Average Diameter (nm)	PDI	DLS Histogram (Intensity%)
	<100 nm	100–200 nm	>200 nm			
NBW-1	N.D.	N.D.	100 ± 0	920 ± 504 ^ab^	1.0 ± 0.0	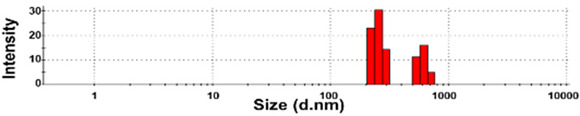
NBW-2	N.D.	N.D.	100 ± 0	513 ± 9 ^ab^	0.2 ± 0.0	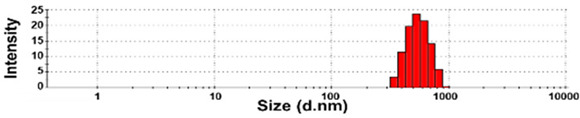
NBW-3	100 ± 0	N.D.	N.D.	130 ± 43 ^a^	1.0 ± 0.0	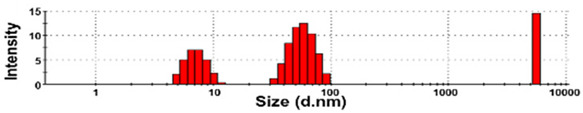
NBW-4	N.D.	N.D.	100 ± 0	1181 ± 397 ^b^	0.9 ± 0.1	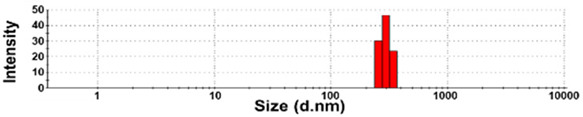
NBW-5	N.D.	N.D.	100 ± 0	1244 ± 105 ^b^	0.9 ± 0.1	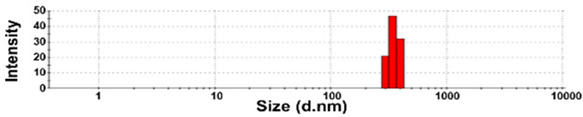
NBW-6	N.D.	N.D.	N.D.	N.D.	N.D.	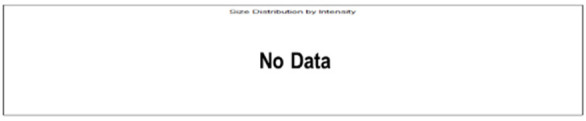
NBW-7	14 ± 19	10 ± 17	76 ± 21	763 ± 525 ^ab^	0.9 ± 0.1	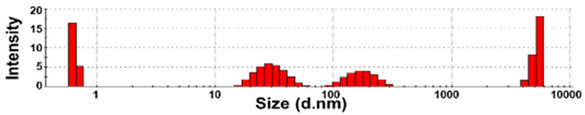
SW-1	100 ± 0	N.D.	N.D.	74,967 ± 42,928 ^c^	0.9 ± 0.2	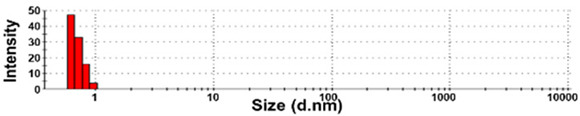
SW-2	100 ± 0	N.D.	N.D.	193,933 ± 69,743 ^d^	1.0 ± 0.0	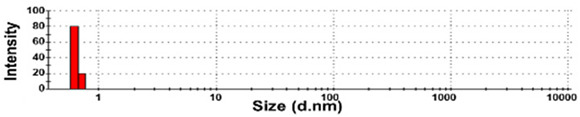

^1,a,b,c,^ and ^d^ indicate significant differences among NBWs and conventional SWs (*p* < 0.05). NBWs, nanobubble waters; SWs, sparkling waters; PDI, polydispersity index; DLS, dynamic light scattering; N.D., not detectable.

**Table 2:** Particle fractions, hydrodynamic diameters, and surface charges of bubbles in NBWs or in conventional SWs ^1^.

Sample	Fraction (Number%)			Z-Average Diameter (nm)	PDI	DLS Histogram (Intensity%)	Zeta Potential (mV)
	<100 nm	100–200 nm	>200 nm				
NBW-1	N.D.	39 ± 31	61 ± 31	422 ± 29 ^c^	0.5 ± 0.2	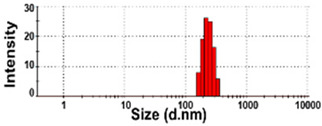	−20 ± 2 ^a^
NBW-2	N.D.	N.D.	100 ± 0	454 ± 33 ^c^	0.2 ± 0.0	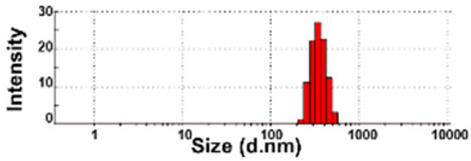	−8 ± 0 ^cd^
NBW-3	100 ± 0	N.D.	N.D.	132 ± 53 ^a^	0.9 ± 0.1	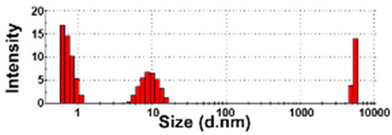	−10 ± 2 ^c^
NBW-4	N.D.	N.D.	100 ± 0	732 ± 51 ^d^	0.2 ± 0.1	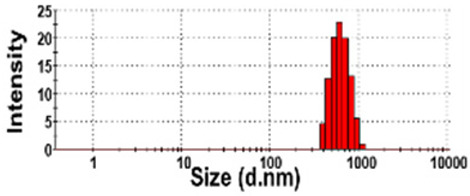	−6 ± 2 ^cd^
NBW-5	N.D.	13 ± 14	87 ± 14	386 ± 39 ^c^	0.3 ± 0.1	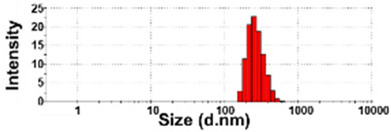	−8 ± 2 ^cd^
NBW-6	100 ± 0	N.D.	N.D.	18 ± 6 ^b^	1.0 ± 0.0	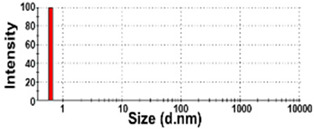	−5 ± 1 ^d^
NBW-7	N.D.	5 ± 5	95 ± 5	369 ± 30 ^c^	0.4 ± 0.2	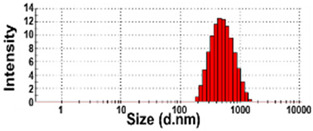	−15 ± 2 ^b^
SW-1	100 ± 0	N.D.	N.D.	35,820 ± 7653 ^e^	1.0 ± 0.0	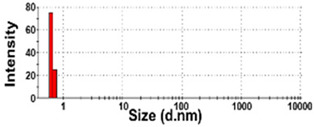	−0 ± 0 ^e^
SW-2	100 ± 0	N.D.	N.D.	61,587 ± 33,772 ^e^	1.0 ± 0.0	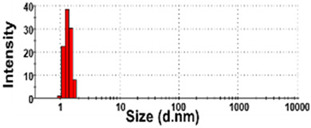	−0 ± 1 ^e^
DW	100 ± 0	N.D.	N.D.	219 ± 10 ^a^	1.0 ± 0.0	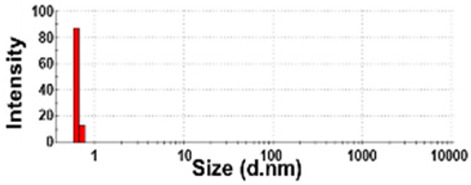	N.D.

^1,a,b,c,d^ and ^e^ indicate significant differences among NBWs and conventional SWs (*p* < 0.05). Dynamic light scattering and electrophoretic light scattering measurements were performed at 2 and 25 °C, respectively. NBWs, nanobubble waters; SWs, sparkling waters; PDI, polydispersity index; DLS, dynamic light scattering; N.D., not detectable; DW, distilled water.

**Table 3:** Particle fractions, hydrodynamic diameters, and surface charges of nanobubbles in NBWs after storage for 6 months ^1^.

Sample	Fraction (Number%)			Z-Average Diameter (nm)	PDI	DLS Histogram (Intensity%)	Zeta Potential (mV)
	<100 nm	100–200 nm	>200 nm				
NBW-1	N.D.	4 ± 6	96 ± 6	601 ± 85 ^d^	0.7 ± 0.1	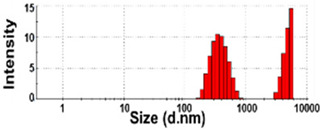	−8 ± 1 ^c^
NBW-2	N.D.	N.D.	100 ± 0	402 ± 14 ^c^	0.2 ± 0.0	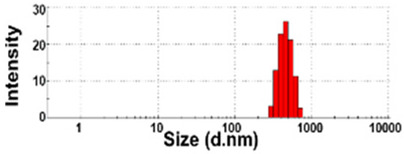	−8 ± 2 ^c^
NBW-3	100 ± 0	N.D.	N.D.	193 ± 92 ^b^	0.9 ± 0.1	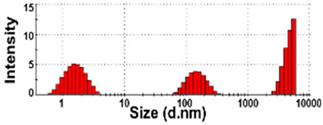	−1 ± 1 ^a^
NBW-4	N.D.	N.D.	100 ± 0	907 ± 238 ^d^	0.9 ± 0.1	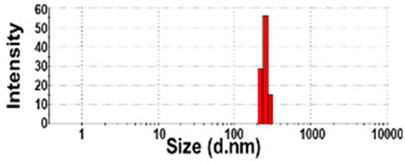	−3 ± 1 ^b^
NBW-5	N.D.	6 ± 6	94 ± 5	358 ± 103 ^c^	0.3 ± 0.1	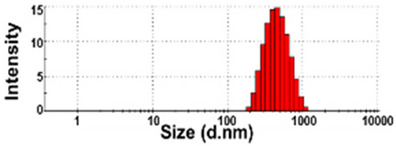	−7 ± 1 ^c^
NBW-6	100 ± 0	N.D.	N.D.	19 ± 5 ^a^	1.0 ± 0.1	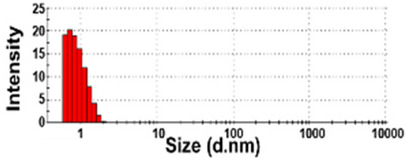	−1 ± 0 ^a^
NBW-7	8 ± 14	46 ± 45	46 ± 48	414 ± 115 ^cd^	0.9 ± 0.2	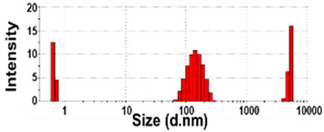	−6 ± 2 ^bc^

^1^_,_^a,b,c,^ and ^d^ indicate significant differences among NBWs (*p* < 0.05). Dynamic light scattering and electrophoretic light scattering measurements were performed at 2 and 25 °C, respectively. NBWs, nanobubble waters; PDI, polydispersity index; DLS, dynamic light scattering; N.D., not detectable.

**Table 4:** Particle fractions, hydrodynamic diameters, and surface charges of TD-NPs and conventional BPs at 25 °C ^1^.

Sample	Fraction (Number%)			Z-Average Diameter (nm)	PDI	Zeta Potential (mV)
	<100 nm	100–200 nm	>200 nm			
NP-1	N.D.	N.D.	100 ± 0	1771 ± 17 ^cd^	0.7 ± 0.3	−21 ± 1 ^d^
NP-2	N.D.	N.D.	100 ± 0	800 ± 30 ^b^	0.2 ± 0.1	−35 ± 0 ^b^
NP-3	N.D.	N.D.	100 ± 0	1756 ± 304 ^cd^	0.7 ± 0.1	−27 ± 1 ^c^
NP-4	N.D.	N.D.	100 ± 0	1198 ± 65 ^c^	0.2 ± 0.0	−31 ± 1 ^bc^
NP-5	N.D.	N.D.	100 ± 0	1403 ± 232 ^c^	0.2 ± 0.1	−32 ± 1 ^b^
NP-6	93 ± 11	6 ± 11	1 ± 0	327 ± 8 ^a^	0.5 ± 0.1	−20 ± 0 ^d^
NP-7	N.D.	N.D.	100 ± 0	2148 ± 376 ^d^	0.3 ± 0.3	−14 ± 3 ^e^
BP-1	N.D.	N.D.	100 ± 0	1246 ± 126 ^c^	0.3 ± 0.2	−27 ± 3 ^c^
BP-2	N.D.	N.D.	100 ± 0	1788 ± 53 ^cd^	0.2 ± 0.0	−47 ± 2 ^a^

^1,a,b,c,d,^ and ^e^ indicate significant differences among TD-NPs and conventional BPs (*p* < 0.05). TD-NPs, top-down-approach-produced nanoparticles; BPs, bulk-sized particles; PDI, polydispersity index; N.D., not detectable.

## Data Availability

The data presented in this study are available in the article and Supplementary Materials.

## Supplementary Materials

The following are available online at https://www.mdpi.com/article/10.3390/foods10092020/s1, Table S1: Major components of NBWs, conventional SWs, TD-NP, and conventional BP products.
