# Supplementary material for: Characterization and Determination of Nanoparticles in Commercial Processed Foods
Source: Foods. 2021 Aug 28;10(9):2020. doi: 10.3390/foods10092020 (PMC8465140; doi:10.3390/foods10092020)
Supplement: Supplementary file 1 [file foods-10-02020-s001.zip › foods-1339244-SI.pdf]

foods

Supplementary Materials

Characterization and Determination of Nanoparticles in Commercial Processed Foods

Jin Yu, Ye-Rin Jeon, Ye-Hyun Kim, Eun-Been Jung and Soo-Jin Choi *

Division of Applied Food System, Major of Food Science & Technology, Seoul Women's University, Seoul 01797, Republic of Korea; ky5031@swu.ac.kr (J.Y.); yrjeon0715@swu.ac.kr (Y.-R.J.); ye4978@swu.ac.kr (Y.-H.K.); ebe2@swu.ac.kr (E.-B.J.)
* Correspondence: sjchoi@swu.ac.kr; Tel.: +82-2-970-5634; Fax: +82-2-970-5977

Table S1. Major components of NBWs, conventional SWs, TD-NP, and conventional BP products. [1]

NBWs		TD-NP products	
**Sample**	**Major Components**	**Sample**	**Major Components**
NBW-1	Water and hydrogen (hydrogen nanobubble)	NP-1	Red ginseng
NBW-2	Water and hydrogen (hydrogen nanobubble)	NP-2	Chaga mushroom
NBW-3	Water and hydrogen (hydrogen nanobubble)	NP-3	Soybean and granola
NBW-4	Water (oxygen nanobubble)	NP-4	Ginkgo biloba
NBW-5	Water and oxygen (oxygen nanobubble)	NP-5	Grape seed
NBW-6	Water and oxygen (oxygen nanobubble)	NP-6	Lactic acid bacteria
NBW-7	Water (ozone nanobubble)	NP-7	Oyster shell calcium
**Conventional SWs**		**Conventional BP products**	
**Sample**	**Major components**	**Sample**	**Major components**
SW-1	Water and carbon dioxide	BP-1	Red ginseng
SW-2	Water and carbon dioxide	BP-2	Chaga mushroom

[1] NBWs, nanobubble waters; SWs, sparkling waters; TD-NP, top-down approach produced nanoparticle; BP, bulk-sized particle.
